# Deciphering the generating rules and functionalities of complex networks

**DOI:** 10.1038/s41598-021-02203-4

**Published:** 2021-11-25

**Authors:** Xiongye Xiao, Hanlong Chen, Paul Bogdan

**Affiliations:** Ming Hsieh Department of Electrical and Computer Engineering, University of Southern California, Los Angeles, CA 90007 USA

**Keywords:** Complex networks, Applied physics

## Abstract

Network theory helps us understand, analyze, model, and design various complex systems. Complex networks encode the complex topology and structural interactions of various systems in nature. To mine the multiscale coupling, heterogeneity, and complexity of natural and technological systems, we need expressive and rigorous mathematical tools that can help us understand the growth, topology, dynamics, multiscale structures, and functionalities of complex networks and their interrelationships. Towards this end, we construct the node-based fractal dimension (NFD) and the node-based multifractal analysis (NMFA) framework to reveal the generating rules and quantify the scale-dependent topology and multifractal features of a dynamic complex network. We propose novel indicators for measuring the degree of complexity, heterogeneity, and asymmetry of network structures, as well as the structure distance between networks. This formalism provides new insights on learning the energy and phase transitions in the networked systems and can help us understand the multiple generating mechanisms governing the network evolution.

## Introduction

From genomic^1,2^, proteomic^3,4^, metabolic^5,6^, and physiologic^7^ networks to microbial communities^8^, neural^9,10^, social^11,12^, material^13^, and technological^14^ systems, we encounter complex interdependent networks with distinct dynamics yet unknown mechanisms of evolution and self-optimization. Although network science provides some metrics such as centrality and clustering coefficient that can shed light on localized connectivity patterns, we lack mathematical tools to quantify the degree of heterogeneity and complexity in the generating rules of a complex network that is the result of unknown evolving mechanisms. To better understand the growth, topology, dynamics, multiscale structures, and functionalities of complex networks, we need advanced analysis algorithms that can comprehensively learn the network structure and determine the relationships between scale-dependent network metrics and their functionalities.

Much of our understanding of dynamic complex networks, their structural features, generating rules, and emerging functionalities rests on addressing the following questions: How can we learn the topology, multiscale structures, and generating rules of complex networks? How can we quantify the degree of heterogeneity and complexity in the generating rules of a complex network? How can we efficiently quantify the distance between two networks? How can we link the functionalities and structure of a network? To address these research questions and break the bottleneck in the field of network analysis, we construct a node-based multifractal analysis (NMFA) framework. The traditional multifractal analyses (MFA)^15–19^ of complex networks are used to observe the self-similarity of some complex networks at different scales based on renormalization procedures. However, when it comes to some real networks and small-world networks, the traditional MFA method fails to capture the structural scaling dependence (see Supplementary Note 4), which limits its applications. In contrast, we propose a universal and reliable framework that can be used in all kinds of networks to decipher their multifractal structures and mine the heterogeneity and complexity of them based on the multifractal analysis. Inspired by the box-covering method^15^, we first propose the box-growing method and the node-based fractal dimension (NFD) to quantify the spatial dimension of a network as it expands or grows from each node, which can be used to learn the generating rules of the network from the nodes. Based on the NFD, our proposed NMFA method deciphers the generating rules of the network by applying the box-growing method on each node to capture the multiple growth rules that can be specific to a network (which can be characterized by the higher-order relationship between the box mass and the box size) and combining these features into the multifractal analysis.

The NMFA can distinguish the various structural behaviors by a higher-order distortion exponent and reveal the evolution of the mechanism behind the dynamic networks. Relying on the NMFA concept, we propose two novel network metrics: (i) a network structure distance to quantify the structural distance between networks; (ii) a multifractal asymmetry metric to measure the multiscale structural asymmetry of a network. We show that the NMFA framework can decipher the generating rules of networks and reveal the relationship between network structures and functionalities. The NMFA framework proves to be a powerful and stable tool to investigate a variety of networks and helps us comprehensively learn their topological features and higher-order connectivity patterns, as well as reveal the structure, dynamics, generating rules and functionalities of the network and their interrelationship, opening a new chapter in network science.

## Results

### NMFA quantifies the complexity and heterogeneity of network formation

Deciphering the heterogeneity and complexity of unweighted and weighted networks (e.g., quantifying the richness in terms of higher-order connectivity patterns and number of graph generation rules) requires new advanced mathematical tools. Consequently, we introduce the node-based fractal dimension (NFD) and the node-based multifractal analysis (NMFA) concepts.

**Figure 1:**
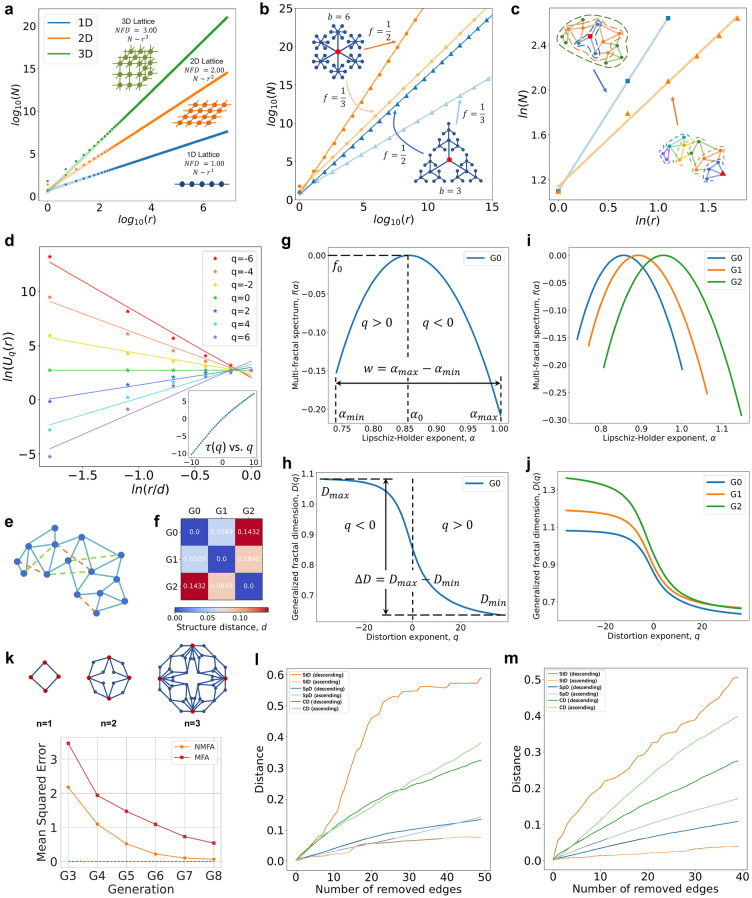
NFD and NMFA quantify the network structure and reveal their generating rules. (**a,b**) The power-law dependence between the number of nodes (*N*) in the box and the box radius (*r*) centered on the single node quantifies the spatial expansion of the network structure, revealing the generating rules. (**a**) The node in the infinite unweighted networks. (**b**) The origin node of the weighted Sierpinski fractal networks. (**c**) The box-growing method of two different nodes (marked as red square and red triangle, respectively) in the artificial network G0. (**d**) The logarithmic relationship between the partition function and the observation scale, and the non-linear relationship between the mass exponent $\tau (q)$ and *q*. (**g,h**) the multifractal spectrum (**g**) and the generalized fractal dimension (**h**) of the network G0. (**e**) The network examples. G0 is the network mentioned above composed of blue nodes and edges, we add three edges (orange dashed line) to form G1 and continue to add three edges (green dashed line) to form G2. (**f**) The structure distances between G0, G1, and G2. (**i,j**) The comparisons of the multifractal spectra **(i)** and the generalized fractal dimensions (**j**) between G0, G1, and G2. (**k**) The mean squared error (MSE) between the mass exponent distributions calculated by MFA and NMFA and the analytical mass exponent distribution of the (u, v)-flower network (u = 2, v = 2). (**l,m**) Three distance methods (i.e., structure distance, spectral distance and correlation distance) comparison using the Ollivier–Ricci curvature and the edge betweenness centrality.

The NFD of a network node represents its topological properties (see “Methods” section where the NFD quantifies the spatial expansion of a network structure centered on a node). The proposed box-growing method (see “Methods” section) on a single node can be viewed as describing the growth of the network from this node by considering it as the origin node of the network. We consider the fractal dimension associated as a parameter to a node, and the value of NFD represents the topological feature of the node. To understand the meaning of NFD intuitively, we provide some basic examples (see Fig. 1a,b). In infinite unweighted lattice networks, the NFD represents the power-law exponent of the interdependence between the mass distribution and the observation scale, and so quantifies the topological dimension (see Fig. 1a). Under the growing observation scales, the NFD of a node equals the spatial dimension in which the network expands, reflecting the generating rule of the network from the node; higher NFD means higher spatial dimension and higher space-filling capacity. In contrast, the introduction of weights in weighted networks influences their geometric properties. The NFD of a weighted network reflects the relationship between the weights and the higher-order connectivity patterns, revealing the geometric properties of weights. To better illustrate this enhanced complexity, we consider the weighted Sierpinski fractal networks constructed according to several copies (*b*) and scaling (*f*) factors. Figure 1b shows the NFD of the origin node for four Sierpinski networks with different growth rules: $b=3, f=1/3; b=3, f=1/2; b=6, f=1/3$; and $b=6, f=1/2$, respectively. They exhibit monofractal behavior with an $NFD = ln(b)/ln(1/f)$, which can be characterized by a single generating rule.

For most real networks, we lack knowledge of the number and type of generating rules. Figure 1c shows two nodes in the network G0 exhibiting different fractal exponents; thus, we cannot use a single NFD value to describe the complexity of the entire network. One can wonder whether we can characterize and mine the higher-order connectivity complexity in heterogeneous networks that are the result of multiple interacting generating rules. Consequently, we propose the NMFA to characterize the multifractal behaviors of the network under different observation scales and introduce the distortion exponent *q* to distinguish the details of different fractal features of the network. Next, we estimate the partition function under different *q* values (see Fig. 1d), and through the Legendre transformation, we determine the multifractal spectrum $f(\alpha )$ as a function of Lipschitz–Holder exponent $\alpha$ (see Fig. 1g) and the generalized fractal dimension (see Fig. 1h).

In the multifractal spectrum, the Lipschitz–Holder exponent $\alpha$ is a measure of singularity; in general, a higher $\alpha$ indicates a higher degree of regularity. Here, $\alpha$ represents the network structural singularity, revealing the fractal complexity of the network structure. The multifractal spectrum $f(\alpha )$ encodes the set of structures showing similar $\alpha$ values. We define $\alpha _{0}$ as the value for which $f(\alpha )$ attains maximum representing the most common singularity. In this way, a network can be considered as a set of multiple fractal structures characterized by the multifractal spectrum, where $\alpha$ represents the complexity and $f(\alpha )$ shows the distribution of the singularities. The width of the spectrum $(w=\alpha _{max}-\alpha _{min})$ reflects the degree of structural heterogeneity; the wider the spectrum, the more heterogeneous the network. Moreover, we use the $\alpha _{0}$ to represent the degree of structural complexity, meaning that the higher $\alpha _{0}$ is, the more complex the network. Equivalently, the generalized fractal dimension *D*(*q*) shows the distribution of dimensions under different distortion exponent *q*; when $q>0$, the larger probability measures will be amplified, while for $q<0$ the smaller probability measures will dominate in the partition function (see “NMFA” in “Methods” section). Compared with the previous MFA methods, NMFA mines the multifractal structures of the network more comprehensively and provides a more accurate estimation (see Fig. 1k, Supplementary Note 4). Being able to quantify the fractal dimension of network structures at different scales allows us to calculate the divergence between the generalized fractal dimension *D*(*q*) for two networks and measure the topological dimension distance between them, thus we propose the structure distance *d* to define the structural distance between two networks (see “Network structure distance” in “Methods” section).

To show how the NMFA can quantify the structural complexity and heterogeneity and capture the structural variation of networks, we consider a simple example in Fig. 1e, where we add edges to the original network G0 to form G1 and G2. Figures 1i,j show the comparisons of these three networks in terms of the multifractal spectrum $f(\alpha )$ and the generalized dimension *D*(*q*). The addition of edges makes the network more complex and heterogeneous as the multifractal spectrum moves to the right (the Lipschitz–Holder exponent $\alpha$ becomes higher) and becomes wider, and the generalized fractal dimension becomes higher. We can learn from the multifractal spectrum that the degrees of complexity of the networks are 0.8586, 0.8953, and 0.9576, respectively; the degrees of heterogeneity of the networks are 0.2604, 0.2938, and 0.3412, respectively. Figure 1f illustrates the structure distance between these networks, where we can numerically learn the structural distance between each pair of networks. G0 and G2 show the largest structure distance. We can see that the NMFA can capture and quantify the small structural changes in the network. In the following application of NMFA for complex networks, we also show that the NMFA is a powerful tool to quantify the complexity and heterogeneity of real complex networks and capture the structural changes.

The structure distance *d* based on NMFA is designed to detect and quantify the differences in structure of networks. We find that the other methods can only capture the edit distance between the networks, but not the structural distance (see Supplementary Note 5). To better emphasize and distinguish the difference between the edit distance and the structural distance, we conduct an edge deletion experiment^20^. In the experiment, we delete edges in the order dictated by their importance quantified through an optimal transport theory inspired strategy. The deletions of different edges have obviously different effects on the structure of the network. The more important the edge is to the network, the greater the effect on the network after deleting that edge. To quantify the importance of an edge, we consider the Ollivier–Ricci curvature (ORC)^21,22^ and the edge betweenness centrality^23,24^ metrics. The ORC extends the curvature notion to networks and measures the deviation of a geometric object from being flat (Euclidean plane). It reveals how the geodesic path converges (ORC is negative) or diverges (ORC is positive). More precisely, an edge with a negative ORC represents a “bottleneck” or essential “bridge” between two parts of the network, while an edge with a positive ORC denotes a nonessential “bridge” for the transport process of the network. Hence, the ORC quantifies the importance of the edge. The edge betweenness centrality captures the number of the shortest paths that pass through that edge. Thus, an edge with a high edge betweenness centrality value represents a “bridge” between two communities of the network, and its removal can affect the information transfer among nodes along the shortest paths.

We first assign the importance to the edges of the network by measuring the ORC or the edge betweenness centrality, then we remove edges in a specific order, i.e., in descending/ascending importance order, and finally we calculate the distance of the current network from the original network. More precisely, we use the Watts–Strogatz model to generate a small-world network with 200 nodes and remove edges according to the ascending or descending order of their importance, which is measured by the values of the ORC (the result is shown in Fig. 1l) and the edge betweenness centrality (the result is shown in Fig. 1m). As shown in the results, the structure distance presents a clear distinction between two removal orders, i.e., ascending order and descending importance order, which means the network with more important edges removed will have a higher distance from the original network. In contrast, the spectral distance and the correlation distance do not show a clear distinction between the two ascending and descending order removal of edges. It even appears that the network distance generated by the deletion of unimportant edges is greater than the deletion of important edges. This is because these two methods exhibit a behavior closer to the edit distance, i.e., only measure the number of operations that transform each other between two networks (see Supplementary Note 5). This experiment proves that the proposed structure distance has unique advantages over prior distances, and has the efficacious capability to detect and quantify the differences in the structure of networks.

### NMFA can identify the dynamic transitions in a complex network with unknown evolving interacting generating rules

**Figure 2:**
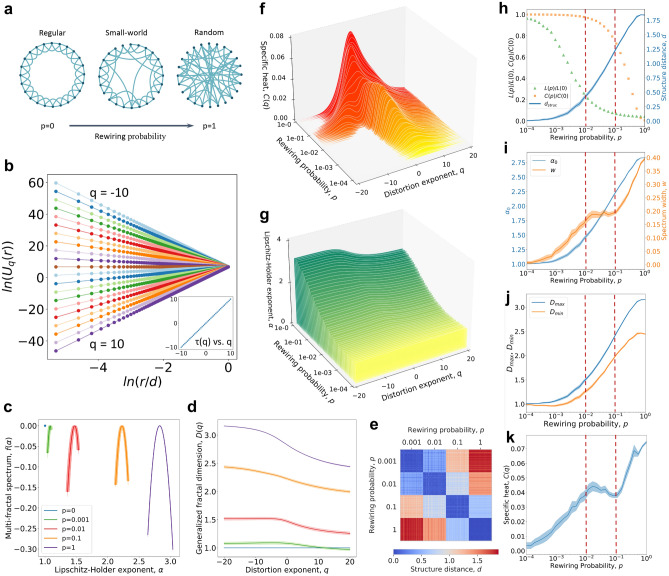
The evolution of the Watts–Strogatz network. (**a**) The mechanism of the Watts–Strogatz network, which starts from a regular network. As the rewiring probability increases, the randomness increases, the network gradually becomes random. (**b**) The logarithmic relationship between the partition function and the scale, and the linear relationship between the mass exponent $\tau (q)$ and *q* for the initial regular network with the rewiring probability $p=0$. (**c–e**) The multifractal spectrum (**c**) and the generalized fractal dimension (**d**) of 1000-node Watts–Strogatz networks with $p=0, 0.001, 0.01, 0.1, 1$ (for each *p*, the analysis is repeated 100 times). (**e**) The structure distance between the 400 networks with $p=0.001, 0.01, 0.1, 1$ (we generate 100 networks randomly for each *p*). (**f,g**) The variations in the distribution of the specific heat (**f**) and the Lipschitz–Holder exponent (**g**) when *p* increases from 0 to 1 (we take 50 sets of *p* at equal intervals, repeat 100 times for each *p* and use the mean value). (**h–k**) As *p* increases from 0 to 1, the evolution of the structural distance from the initial regular network (**h**); the degree of the complexity $\alpha _{0}$ and the heterogeneity *w* (**i**); the measure of fractal dimension $D_{max}, D_{min}$ (**j**); and the measure of specific heat $C(q)$ (**k**). The solid lines in figures represent the mean value while the shades being the 99% confidence interval.

Knowing that NMFA can quantify the complexity and heterogeneity in the generating rules, and capture the structural changes of the network, we infer that it can be used to observe the evolution of the structure and generating rules of the complex dynamic networks. To best illustrate this process, we consider a dynamic edge rewiring process in a Watts–Strogatz (WS) network model^25^ whose topology evolves from a regular to a random graph. Simply speaking, we start with a regular lattice network (consisting of 1000 nodes and 2000 edges) and gradually transit to a small-world and lastly to a random network (Erdős–Rényi network) by rewiring edges according to an increasing rewiring probability (see Fig. 2a). During this transition, the size of the WS network does not change (i.e., the number of nodes and edges does not change), but the structures and the generating rules of the network evolve.

In the initial regular lattice network, each node has identical property, obeying the same generating rule. The mass distribution *M*(*r*) and the observation scale *r*/*d* have a linear relationship and the NFD of each node equals 1, representing a linear topology and the linear generating rule. As shown in Fig. 2b, there is a strict power-law dependence between the partition function $U_{q}(r)$ and the observation scale *r*/*d*, leading to the strict linear relationship between the mass exponent $\tau (q)$ and the distortion exponent *q*, which indicates a monofractal behavior. The regularity of the initial network (characterized by the linear generating rule) is broken by the edge rewiring process (i.e., increasing rewiring probability *p*) and more randomness is introduced (i.e., the network becomes more complex and heterogeneous). This is best reflected in Fig. 2c which shows that the monofractal like behavior at $p=0$ (shown as a single point in the multifractal spectrum) transits to multifractal behavior, as *p* increases, the spectrum moves to the right and the width of the multifractal spectrum increases. The complexity and the heterogeneity of the dynamic network also increase the spatial dimension or the space-filling capacity of the network; this is reflected in the generalized fractal dimension becoming higher and wider from a horizon (representing the monofractality) (see Fig. 2d). In the experiment, we generate the WS network 100 times randomly for each $p = 0.001; 0.01; 0.1; 1$, and show the structure distances between these 400 networks in Fig. 2e. The networks generated under different *p* values show the obvious boundaries of the network structures, implying different generating mechanisms. We conclude that the rewiring process introduces more complex patterns, increases the difference between the fractal structures, and constantly breaks the regularity of the networks, leading to higher complexity and heterogeneity. Through the numerical values of $\alpha _{0}$ and *D*(*q*), we can identify that the WS network transitions from linear topology (the values are around 1) to cubic topology (the values are around 3) as *p* increases from 0 to 1, implying a transition in the generating rules.

#### Thermodynamics-inspired characterization of networks

Can we learn the energy and phase transition of a network from NMFA? We conjecture that the NMFA can provide a thermodynamics inspired representation of a network, or we can view the network as a thermodynamic system. The mass exponent $\tau$, the Lipschitz–Holder exponent $\alpha$, the derivative of $\alpha$, and the distortion exponent *q* are analogous to the free energy, the energy, the specific heat, and the temperature in thermodynamic systems^26–28^, respectively. The phase of a thermodynamic system has uniform physical properties. The phase transition means that certain properties change discontinuously under a critical external condition. Consequently, we can investigate the phase transitions of a network by studying whether the network “energy” $\alpha$ undergoes a sharp jump near a critical “temperature” $q_{c}$. In the $\alpha$ distribution under *q*, the “energy” $\alpha$ has a large fluctuation near $q_{c}$, which can be reflected by the peak of the specific heat $C(q_{c})$. For *q* values below $q_{c}$, the “free energy” $\tau$ is dominated by the largest energy term $\alpha _{max}$, which indicates a network structure with high singularity index. After the large energy fluctuation near $q_{c}$, the dominant part becomes the small energy term $\alpha _{min}$. Therefore, the specific heat can be also considered as a measure of the difference between high and low singularity in the network structures.

Here, we consider the WS network as a thermodynamic system and the rewiring process as a dynamic transition from order to chaos (see Fig. 2a). By rewiring the edges in the WS network, Fig. 2f,g show the continuous variations in the distributions of the specific heat *C* and the energy $\alpha$ under the distortion exponent. The $\alpha$ and *C* become higher with increasing rewiring probability *p*, indicating the energy and the specific heat of the networked system increase with a higher degree of randomness. One interesting phenomenon is that when $0.01< p < 0.1$, the specific heat distribution of the network has two different peaks, showing there are two phase transitions with different critical distortion exponent values. This means that the system can be seen as a combination of two sub-networks obtained according to two generating mechanisms with different phase transition points, which implies a differentiation in the network structure with an obvious boundary. It is worth noting that the small-world property of the WS network is displayed in this region.

#### Special multifractal behaviors in the “small-world” region

The small-world property describes a phenomenon in which despite the large number of nodes in the network, starting from one node, it only takes a few steps to reach any other node, also known as six degrees of separation^29^. In the WS model, the newly constructed networks which have high clustering coefficients (close to the initial lattice network) and low path lengths (close to the final random network) are considered to be the small-world networks^25^. To study the small-world property regime, we show a continuous evolution of the network structural features as a function of the rewiring probability *p* (see Fig. 2h–k). In Figure 2h, while increasing the rewiring probability *p* from 0 to 1, there is a regime in which the networks are highly clustered yet have small path lengths, representing the small-world property; we mark this small-world regime within two red dashed lines (i.e., $0.01<p<0.1$).

Corroborating Figures 2h–k, we observe that the small-world regime is characterized by a stable multifractal spectrum width and a decreasing specific heat peak. We also observe from Fig. 2f that the transition in the small-world regime (where lattice and Erdős–Rényi like structures coexist) is characterized by a shift in the specific heat distribution from a pronounced local peak to one that has a lower peak and is more evenly dispersed. Alternatively, in this transition, the heterogeneity does not change, the peak value of the specific heat decreases but the structure distance from the initial network, the complexity, and the dimension increase (since the Erdős–Rényi structure has higher complexity and dimension). When *p* is around 0.1, as the mesoscale regular and Erdős–Rényi structures gradually merge, the boundary between the structures of the two mechanisms disappears (the two peaks of the specific heat distribution merged into one), and the network evolves from a small-word network to a random network. When $p > 0.1$, the WS network loses the small-world property and becomes more complex and heterogeneous as p increases. Basically, Figure 2i shows that the complexity and the heterogeneity become higher with the increasing *p*. Therefore, the NMFA provides new insights into the small-world networks by indicating the emergence and stability of a phase, as well as quantifying the coexistence of multiple interacting generating mechanisms on an evolving network.

### NMFA reveals the structural asymmetry and the generating rules of real complex social networks

Scale-dependent analysis of complex networks can reveal frequent and rare network motifs with implications for their dynamics and functionality. More precisely, the $q > 0$ amplifies the larger probability measures (i.e., frequent network structures) and encodes their fractal properties in the left part of the multifractal spectrum, while the $q < 0$ magnifies the small probability measures (i.e., rare network motifs) and encodes their fractal characteristics in the right part of the multifractal spectrum. This allows us to quantify the multiscale asymmetry and reveal the multiscale generating rules of networks. To better illustrate the networks structural asymmetry, we consider two networks: (i) a random network generated via Erdős–Rényi model^30^ with a fixed linking probability which leads to the formation of a “thorn” structure (for lower linking probabilities), and (ii) a scale-free network generated via a preferential attachment strategy as in the Barabasi–Albert (BA) model^31^ which means that a node with more connections is more likely to receive new links and leads to the formation of a “clump” structure (see the “Network generation model” in “Methods” section).

**Figure 3:**
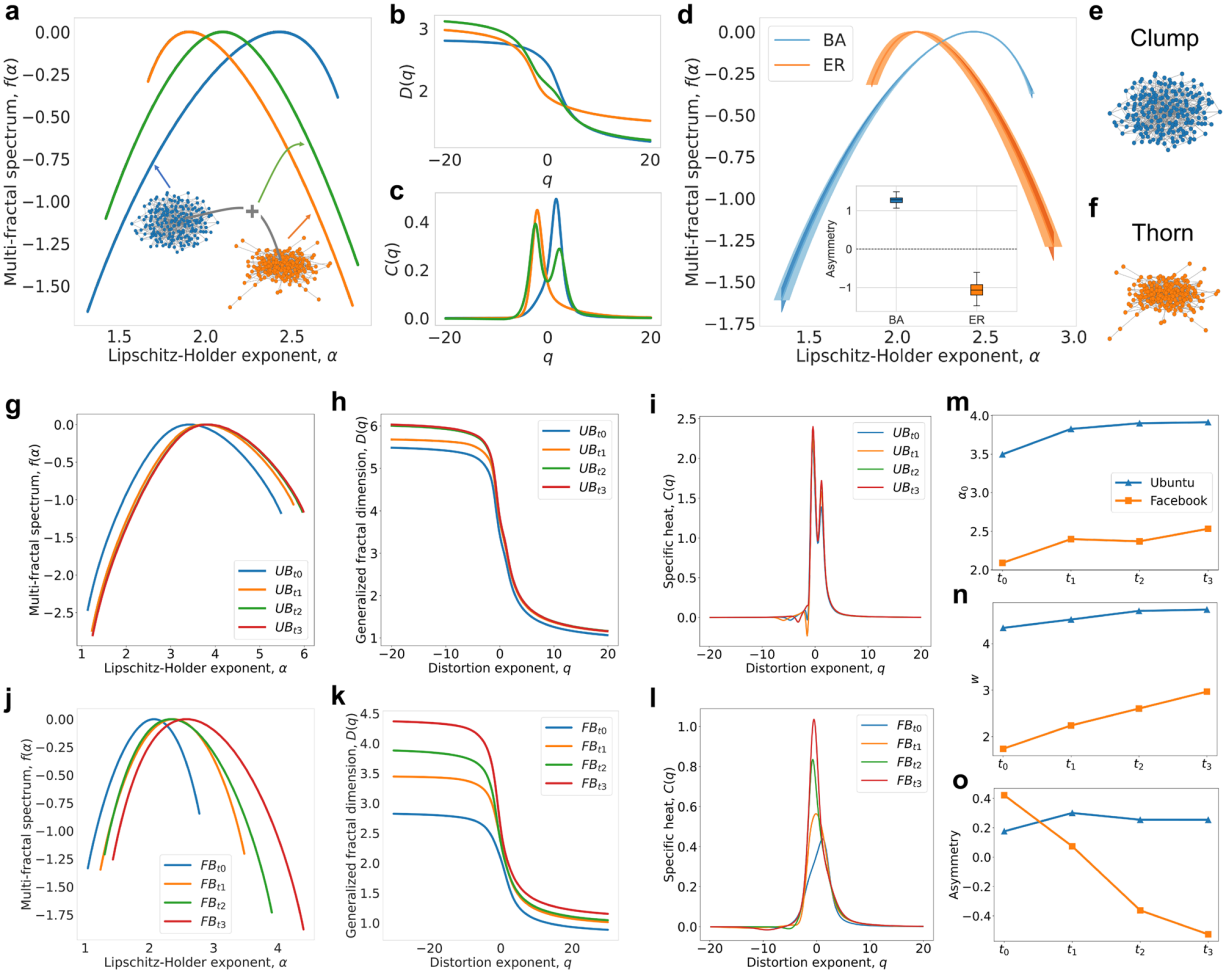
NMFA of real complex social networks. (**a–c**) We use BA and ER models to generate two networks with a similar number of nodes (200) and edges (600), and a merged network of them by linking their highest connected nodes with one edge (**a**). The differences between their multifractal spectra (**a**), generalized fractal dimension (**b**), and specific heat distributions (**c**). The BA, ER, and merged networks are represented as blue, orange, and green respectively. (**d**) We use the BA and ER models to generate 100 networks with a similar number of nodes (200) and edges (600) and measure their multifractal spectra and asymmetry shown in (**d**). The solid lines in the figures represent the mean value while the shades represent the 99% confidence interval. (**e,f**) The different structural features of BA network (clump) (**e**) and ER network (thorn) (**f**). (**g–l**) We use NMFA to show the multifractal features of two typical social networks changing over time. (**g–i**) The variations of the multifractal spectra (**g**), the generalized fractal dimension (**h**), and the specific heat distributions (**i**) of the Ask Ubuntu networks. (**j–l**) The variations of the multi-fractal spectra (**j**), generalized fractal dimension (**k**), and specific heat distributions (**l**) of the Facebook-like Forum networks. (**m–o**) The variation of the degree of complexity (**m**), heterogeneity (**n**), and asymmetry (**o**) of the two social networks for different time stamps.

Exploiting the asymmetry metric (see the “Asymmetry” in “Methods” section), we observe: a network with a positive multifractal spectrum-based asymmetry possesses a “clump” property like the BA network (see Fig. 3e), while a negative asymmetry means the “thorn” structure dominates the network (see Fig. 3f). When the $Asymmetry \approx 0$, it means the network structure is symmetric without a significant dominant structure. Figure 3a shows the multifractal spectra of the ER network, the BA network (the two networks have the same number of nodes and edges), and a merging of them by linking their highest connected nodes with one edge. The merged network shows a combination of two types of structures, one obeys the mechanism of the BA model, the other obeys the mechanism of the ER model. The spectrum of the BA network has a long tail on the left while the spectrum of the ER network has a long tail on the right. The spectrum of the merged network shows a higher heterogeneity as a union of the two spectra, representing the union of the generating rules of the two networks. Figures 3b,c show the merging behavior in *D*(*q*) and *C*(*q*), respectively. It is worth mentioning that the specific heat distribution of the merged network has two peaks, one close to the BA and the other close to the ER, which proves the existence of the boundary between these two different structural mechanisms, exactly confirming the interpretation of the small-world property in WS network that we describe in Fig. 2f. Moreover, we learn critical information about the structural mechanisms from the specific heat distribution by considering the asymmetry: for a phase transition critical value $q_{c} > 0$, the large probability measure dominates the network structure, showing the “clump” mechanism, while for $q_{c} < 0$, the small probability measure dominates the network structure, showing the “thorn” mechanism. With the dominance of large probability measures of the frequent patterns, the “clump” mechanism is considered to be able to support higher, faster, and more robust information transmission.

Figure 3d shows differences in terms of the multifractal spectra and the degree of asymmetry between BA and ER generated networks with 200 nodes and 600 edges and 100 iterations for each configuration. We can see that the network generated under a specific generating mechanism has the stable multifractal spectrum and multiscale asymmetry. The BA and ER networks have different degrees of asymmetry (i.e., the Asymmetry of BA network> 1, showing a “clump” property while the ER network shows a “thorn” property).

To investigate whether real social networks obey a preferential attachment mechanism and show similar asymmetry as the BA model, we analyze two typical social networks (i.e., the Ask Ubuntu^32^ (UB) and Facebook-like Forum^33^ (FB) networks) and quantify their multifractal structures. The UB network is a typical social network, showing how people interact on an ask and answer forum; the number of users in this network has grown from 80,854 in 2013 to 157,220 in 2016. The FB network can be seen as a typical public social community covering a time span from May 2004 to October 2004, with the number of people increasing from 159 to 899. The basic information of the networks is provided in Table 1. As shown in Figures 3g–i, although more users join the UB network and more connections are established with the network size doubled from t0 (year 2013) to t3 (year 2016), the multifractal spectrum, the generalized fractal dimension and the specific heat distribution exhibit small variation from t0 (year 2013) to t1 (year 2014), and almost no change from t1 (year 2014) to t3 (year 2016). This indicates that the UB network has a robust structure in which the mechanism for newly joining users to establish connections is stable. Furthermore, this indicates that the complex network can be simulated and described by a certain mechanism, and the generating rules characterized by the multifractal spectrum are stable. And the multifractal spectra of the UB networks show long tails on the left, representing a “clump” generating mechanism like the preferential attachment mechanism. This is because the UB platform has a large number of core users, which dominate the network. We infer that the new joining users in UB are more inclined to establish connections with multiple core users and can quickly integrate into the network to become new core users, thus the UB network maintains a high information transmission.

In contrast, the multifractal behaviors of the FB network change significantly over time. As the number of members increases, the spectrum shifts to the right and becomes wider. Also, the degree of asymmetry changes (the Asymmetry changes from positive to negative). The generalized dimension and specific heat become higher over time (Fig. 3j–l). The results show that the network structure changes significantly with new members joining in, more complex connections are established between them and the network becomes more complex and heterogeneous. From the change in the asymmetry and specific heat distribution, we can learn that the FB network is initially dominated by core members like a “clump”, and the new member does not join the network following the preferential attachment mechanism but tends to establish a single connection randomly with the member of the network, leading to a change in the multiscale asymmetry. Therefore, the FB network can be seen as an unstable social network that continues to expand. We also compare the multifractal metrics of two networks in Fig. 3m–o showing the UB network has significantly higher complexity and heterogeneity. We conclude that the UB network is a stable social network following the preferential attachment mechanism like the BA model with the high and robust information transmission, while the FB network is a developing network showing the “thorn” structure.

**Table 1:** Basic information of the typical social networks.

Network name	Number of nodes	Number of edges	Average degree	Diameter of the network
Ask Ubuntu (t0)	77,931	232,099	5.96	14
Ask Ubuntu (t1)	110,942	329,853	5.95	13
Ask Ubuntu (t2)	145,656	433,098	5.95	13
Ask Ubuntu (t3)	152,597	453,211	5.94	13
Facebook-like Forum (t0)	159	263	3.31	8
Facebook-like Forum (t1)	384	1216	6.33	7
Facebook-like Forum (t2)	643	3323	10.34	7
Facebook-like Forum (t3)	899	7046	15.68	6

### NMFA reveals the relationship between multifractal behaviors and functionalities of brain networks

**Figure 4:**
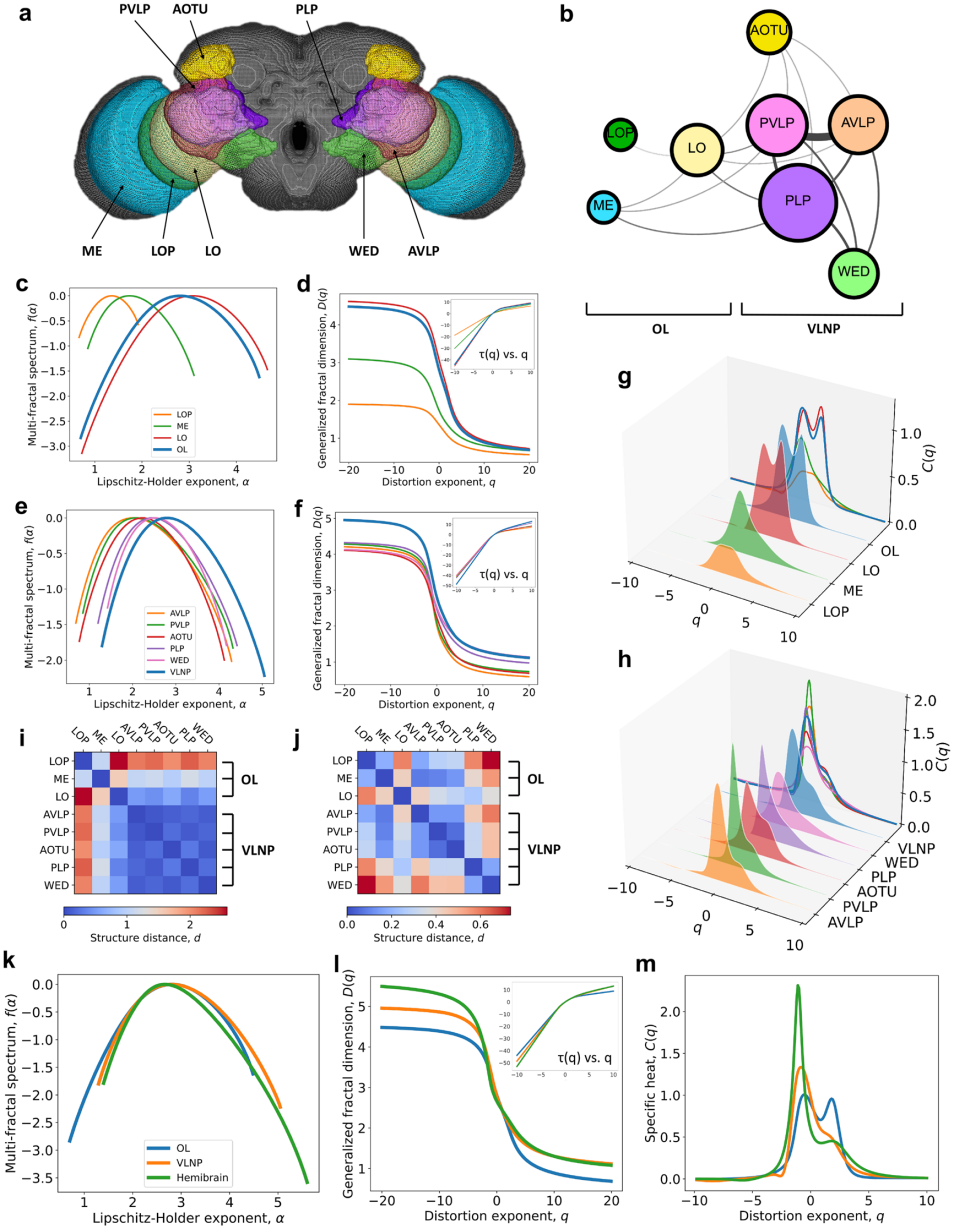
NMFA of the adult Drosophila visual system. (**a**) The composition of the Drosophila visual system. (**b**) The connections between different regions in the Drosophila visual system. The area of the circle characterizes the size of the corresponding brain regions (i.e., number of neurons) and the width of the line between two circles characterizes the connection between the brain regions (i.e., number of neural connections shared by two regions). (**c–h**) The comparisons of the multifractal behavior between the networks in the optic lobe (OL) and the ventrolateral neuropils (VLNP). (**c,d,g**) The comparisons of the multifractal spectra (**c**), the mass exponent (**d**), generalized fractal dimension (**d**), and specific heat distribution (**g**) of the networks in the OL. (**e,f,h**) The comparisons of the multifractal spectra (**e**), the mass exponent (**f**), generalized fractal dimension (**f**), and specific heat distribution (**h**) of the networks in the VLNP. (**i,j**) The structure distance between the regional networks when q < 0 (**i**) and q > 0 (**j**) respectively. (**k–m**) The comparisons of the multifractal behaviors between the OL, VLNP, and the whole hemibrain. (**k**) The multifractal spectrum. (**l**) The generalized fractal dimension and mass exponent distribution. (**m**) The specific heat distribution.

Drosophila melanogaster has been widely studied as a model organism. Its visual system containing about 150,000 neurons^34^ manifests sophisticated functions and highly-differentiated networks. Exploiting our NMFA tool, we analyze the neuronal networks^35^ of two major vision-related regions in the adult Drosophila brain: the optic lobe (OL) and the ventrolateral neuropils (VLNP). The OL, where most visual processing occurs, consists of four parts: the lamina, medulla (ME), lobula (LO), and lobula plate (LOP). Each part implements different functionalities. The VLNP belonging to the central brain represents the next visual processing step after the OL and is divided into five regions (i.e., AVLP, PVLP, AOTU, PLP, and WED) (for details see Supplementary). The neurons of the five regions in VLNP are believed to originate mostly from central brain neuroblasts. Although their functionalities are not yet known, we show that the NMFA can help at decoding their contribution to the functionalities by learning their multifractal behaviors and generating rules. The specific locations of the regions we study in this article are shown in Fig. 4a. The neural networks accessed from the hemibrain dataset of FlyEM Project are belonging to the right hemibrain of the Drosophila. Figure 4b shows the size of these regions (i.e., the number of the neurons) and the connection between the regions (i.e., number of neural connections shared by two regions). The area of the circle represents the size of the region, and the line width between two circles represents the connection strength between two regions.

Figure 4c,d show the multifractal spectra $f(\alpha )$, the mass exponent $\tau (q)$, and the generalized fractal dimension *D*(*q*) of the OL networks. The different OL regions show different multifractal spectra with distinctive support of Lipschitz–Holder exponents which indicates they possess distinctive functionalities. And the LO network exceeds all other OL networks in terms of Lipschitz–Holder exponents support. It can be clearly observed that the order of the degree of complexity and heterogeneity of these regional networks is LO>ME>LOP. The LO and OL networks show similar multifractal behaviors, which means the LO network dominates the network structure of the entire optic lobe. Moreover, we can see from Figure 4b that the LO region connects most regions, being connected to all regions except the WED; consequently, the LO network can be considered as a bridge between the optic lobe and the central brain. The LOP region connects the smallest number of regions (i.e., it only connects to the LO region) implying local functionality. We conclude that the network with more complex functionalities will show a higher degree of complexity and heterogeneity. From the multifractal spectrum, we also notice that the LO network shows a “clump” structure, which implies the high and robust information transmission, enhancing its ability to act as an information transmission bridge between the optic lobe and the central brain.

Unlike the obvious differences in multifractal behaviors between the regions in the OL, Fig. 4e,f show that the neuronal networks in the VLNP region exhibit a more similar multifractality. All of these five networks show a high degree of complexity and heterogeneity (compared with networks in the optic lobe). This implies that the various VLNP networks have similar topological structures and can implement redundant or overlapping complex functionalities. The multifractal spectrum of the entire VLNP network is wider and moves to the right, while its generalized fractal dimension is higher than its sub-networks, indicating a more heterogeneous and complex connection structure. This means that there must be some complex high-order connections among these five regions. Figure 4b shows that there are strong connections between these five regions in the VLNP.

Figures 4g,h show the distributions of the specific heat for the regional networks belonging to the OL and the VLNP, respectively. In the OL network, the LOP, ME, and LO sub-networks show different critical values of the phase transition, which represents different structural mechanisms. The LO and OL networks exhibit two peaks in the specific heat distribution, implying two different structural mechanisms. Moreover, this indicates a boundary between network structures with different mechanisms and possibly two different neuron types or functionalities providing new hypotheses for neuroscience investigations. It is known that the LO neurons can be divided into two categories: the columnar neurons (LC) receiving visual input from 8 to 9 ommatidia; and the tangential and treelike neurons (LT) receiving input from very large visual fields^36^. Thus, we infer that the two structural mechanisms may correspond to the LC and LT, and further neuroscience investigations are needed to prove it. In contrast, from Figure 4j, we can see that the critical values of the phase transition in the VLNP’s networks are very close, implying the regional networks in the VLNP have similar structural mechanisms.

Observing the difference between the generalized fractal dimension distributions of these networks for $q < 0$ and $q > 0$ (see Fig. 4d,f), we conclude that these networks show distinct differences between the frequent and rare structures. To further quantify the structural distances, we calculate the structure distance for $q < 0$ and $q > 0$. Figure 4i shows the structure distance in terms of rare patterns (i.e., for $q < 0$) between the VLNP sub-networks is small, which means the fractal behaviors of the rare network structures are very similar in VLNP sub-networks. In contrast, the structure difference between the sub-networks in OL is larger, especially between the LO and the LOP. For $q > 0$ (see Figure 4j), all the networks show a small structure distance ($d<0.8$), implying similar generating rules for the frequent structures in all the neural networks. It also shows that we can capture and quantify the subtle structure distances between the networks from multiscale structures, providing a basis to further distinguish the networks in terms of functionalities.

Figures 4k–m show the NMFA analysis comparison results among the OL, the VLNP, and the hemibrain. These multifractal results indicate that the structure of the entire hemibrain network is more similar to that of the VLNP. Moreover, these three networks share some common generating rules, which are revealed by the overlapping parts of the multifractal spectra. The hemibrain network shows a higher heterogeneity, a higher generalized fractal dimension, and a similar degree of complexity compared to the VLNP. This implies that there may exist some hidden connectivity patterns between the VLNP and OL that do not change the complexity, and the hemibrain have more diverse functionalities. From Figure 4m, we can see the specific heat distribution of the hemibrain have two peaks: one is an obvious peak with the critical value of phase transition $q_{c}<0$, and a non-obvious peak with $q_{c}>0$. This implies the coexistence of the “thorn” structure mechanism and the “clump” mechanism, and the “thorn” mechanism dominates the network structure.

Therefore, we show that we can use the NMFA analysis to study the brain structure and the regional functionalities from a network perspective. Establishing the connection between the network generating rules and the functionalities of the brain regions can help us predict the functionalities through the network structure and simulate the network with specific functionalities.

## Discussion

In this article, we propose the NFD and the NMFA framework to decipher the topological information and the generating rules embedded in the network structures, based on which we define a general approach to quantify the complexity and heterogeneity of networks. We also propose two novel indicators to measure the structure distance between networks and the multiscale asymmetry of a network structure, which could prove revolutionary in the field of network analysis. In addition, we provide a new insight to consider the network as a thermodynamic system and explain the phase transition of the network structure, which gives us a new perspective to decipher the energy and specific heat of a network, and observe its phase transition through the lens of the specific heat distribution. By studying the phase transition, we can explain the essence behind the small-world property of the Strogatz–Watts model, i.e., the coexistence state of lattice and random sub-network mechanisms. Moreover, our method can open new avenues to investigate the generating rules and functionalities of real networks and comprehensively learn about their multiscale structural characteristics.

By applying the NMFA to two typical social networks, we demonstrate how our framework can quantify the difference in terms of multifractal and asymmetry behavior between stable social networks and developing ones. In addition, the NMFA framework can learn the multifractal behavior of different regions of the adult Drosophila brain network in order to find if there is a relationship between these brain region functions and their structures. We also find the structural differentiation of the lobula (LO) region.

The NFD can reveal the topological feature of each node in a network, allowing us to differentiate the nodes, evaluate their contribution to the overall network functionality, and determine the relationship between the topological structure and other intrinsic properties of the node. More specifically, we experiment on the S. cerevisiae protein–protein interaction network^4^ and prove that NFD has an obvious strong ability to differentiate nodes and reveal that the proteins with lower NFD values are more likely to be lethal (see Supplementary Note 2). Therefore, we can use our method to find the relationship between the topological features and the inherent properties of a node.

The NMFA framework is general and applicable to a wide range of application domains for deciphering the information behind the degree of heterogeneity in the generating rules of a network and revealing the relationship between the functionalities and structures of the networks. In complex systems such as cyber-physical systems^37^, the NMFA can be used to decode the complex interactive structures, capture the dynamic variation and detect the hierarchical community structures^38,39^. In cognitive neuroscience, it is believed that the organization of the structural wiring among neurons largely determines the types of cognitive functions in the brain^40,41^. It has been proved that the brain is characterized by heterogeneous patterns of structural connections; through our method, we are able to learn new scale-dependent knowledge about the structural wiring of the brain networks and investigate the relationship between the brain network structures and cognitive functions. Although several efforts are focusing on brain circuits with complex interactive functions such as the action and perception circuits^42^ and the circuits implementing our “space” and “time” perception in the brain^43^, we need more comprehensive and rigorous tools to decipher the multiple mechanisms and heterogeneity of these cognitive processes and their circuit implementation in the brain. We conclude that NMFA can serve this purpose and help us further understand the brain network plasticity mechanisms and unravel the relationship between the functions and network structures. Relying on recent in vivo sensing technology, we can monitor and determine the generating rules behind neuronal networks in different brain regions via the NMFA framework, establish a correspondence between the brain regional function and its multifractal behavior, and possibly identify new design methodologies for next-generation artificial neural networks capable of advanced perception, generalization, and decision making from scarce and noisy observations. Thus, this framework offers the possibility to judge the functionality through the lens of generating rules of the scale-dependent connectivity of a brain network.

## Methods

To capture the topological features and the degree of complexity and heterogeneity in generating rules of complex networks, we propose the box-growing method, the node-based fractal dimension (NFD) and the node-based multifractal analysis (NMFA) framework. Based on our method, the complexity and heterogeneity of the network can be quantified and the fractal structural features of the growth of the networks can be characterized by the node level information.

### Node-based fractal dimension (NFD)

Different from the cluster-growing method^44–46^ and the box-counting method^15,16^ that calculate the dimension of the whole network, we first propose box-growing method to measure the spatial dimension of the expansion or the growth of the network from a certain node which can be considered as the origin node of the network, and we consider the dimension as a parameter of the node. Formally, let us consider the node *i* as the origin node of the network and then treat it as a growing-box with an initial radius equal to 0. Next, we increase the box radius until it covers at least one node in the network; let this radius be $r_{0}$ and the mass distribution (i.e., the number of nodes in the growing-box) be $M(r_{0})$, which is considered as the initial mass of the network generation. We continue to increase the box radius *r* until it covers the entire network; the relationship between the mass distribution *M*(*r*) and the box radius *r* can be characterized as:

1 $$\begin{aligned} \frac{M(r)}{M(r_{0})} \sim \left( \frac{r}{r_{0}}\right) ^{D}, \end{aligned}$$

where *D* is the fractal dimension representing the spatial dimension of the growth of the network centered on node *i*. We design the node-based fractal dimension (NFD) to capture the generating rule (the power law between the mass distribution and the growing-box size) of the network with a certain node as the origin of the network, the NFD of node *i* is defined by:

2 $$\begin{aligned} NFD_{i}=\frac{\ln \left( \frac{M(r)}{M(r_{0})}\right) }{\ln \left( \frac{r}{r_{0}}\right) }. \end{aligned}$$

For the network generated by a single rule, the NFD can be used to characterize the spatial expansion of the network from the origin node, and the value of NFD represents the degree of the complexity of the generating rule. When it comes to heterogeneous networks with multiple generating rules, we need multifractal analysis.

### Node-based multi-fractal analysis (NMFA)

To capture the multiple generating rules and multifractal features of the network structure, we propose the NMFA. Based on the box-growing method, we integrate the fractal features of all nodes in the network. The distortion factor *q* is introduced to distinguish the details of different structural features. We define the probability measure of node *i* as $u_{i}(r)=\frac{M_{i}(r)}{M}$, where *M* is the total mass (e.g., the total number of nodes in the network). The partition function (the sum of the *q*th power of the probability measures) is defined as:

3 $$\begin{aligned} U_{q}(r)=\sum _{1}^M u_{i}(r)^{q}. \end{aligned}$$

There is a power–law relationship between $U_{q}(r)$ and the observation scale $\frac{r}{d}$: $U_{q}(r)\sim (\frac{r}{d})^{\tau (q)}$, where *d* is the radius of the network (maximum box radius) and $\tau (q)$ is the mass exponent and can be calculated as:

4 $$\begin{aligned} \tau (q)=\frac{ln(U_{q}(r))}{ln(\frac{r}{d})}. \end{aligned}$$

Through Legendre transform^47^, the multifractal structural features of the network can be shown by the multifractal spectrum $f(\alpha )$:

5 $$\begin{aligned}&\alpha (q)=\frac{d\tau (q)}{dq}, \end{aligned}$$

6 $$\begin{aligned}&f(\alpha )=q\alpha (q)-\tau (q), \end{aligned}$$

where $\alpha (q)$ is the Lipschiz–Holder exponent. The fractal dimension of the fractal features is captured by the generalized dimension *D*(*q*):

7 $$\begin{aligned} D(q)=\frac{\tau (q)}{q}. \end{aligned}$$

The multi-fractal spectrum and the generalized dimension quantify the inhomogeneity of fractal structures representing multiple generating rules contained in the network. The NFD and NMFA represent strong tools to decipher the complexity and heterogeneity in generating rules of complex networks, as well as capture the structural differences between different networks to detect anomalies.

### Network structure distance

Based on NMFA, we propose a metric distance to quantify the structural distance between two networks. The structural features are captured by the generalized fractal dimension. Networks with distinct structural features have a different distribution of *D*(*q*) under the distortion exponent *q*. Therefore, we can quantify the distance between two networks by calculating the divergence (i.e., the standard deviation between two distributions) in the distribution of the dimensions under *q*. The structure distance between two networks is defined as:

8 $$\begin{aligned} d=\sqrt{\frac{\int _{q_{min}}^{q_{max}}(D_{1}(q)-D_{2}(q))^{2}\,dq}{q_{max}-q_{min}}}. \end{aligned}$$

In this way, we can learn the structure distance between the two networks numerically. Intuitively, the value of structure distance *d* represents the difference between the topological dimensions of the structures of two networks.

### Asymmetry

In the multifractal spectrum, the $q>0$ amplifies the larger probability measures (i.e., frequent network structures) and encodes their fractal properties in the left part of the spectrum, while the $q<0$ magnifies the small probability measures (i.e., rare network motifs) and encodes their fractal characteristics in the right part of the spectrum. This allows us to quantify the multiscale asymmetry of networks. Here, we propose the asymmetry metric as follows:

9 $$\begin{aligned} Asymmetry=\ln \left( \frac{\alpha _{0}-\alpha _{min}}{\alpha _{max}-\alpha _{0}}\right) . \end{aligned}$$

Therefore, the Asymmetry of the network measures the difference between the multiscale structures of the network. When $Asymmetry > 0$, the large probability measures (frequent patterns) dominates the network structure; when $Asymmetry < 0$, the small probability measures (rare patterns) dominates the network structure; when the $Asymmetry \approx 0$, it means the network structure is symmetric without a significant dominant structure.

### Thermodynamics-inspired characterization of networks

Relying on the NMFA concepts, we provide a new insight to view the network as a thermodynamic system. In NMFA, the mass exponent $\tau (q)$, the Lipschitz–Holder exponent $\alpha$, the multifractal spectrum $f(\alpha )$, and the distortion exponent *q* are analogous to the free energy, the energy, the entropy, and the temperature in a thermodynamic system^27^, respectively. Then, the specific heat *C*(*q*) is defined as:

10 $$\begin{aligned} C(q)=\frac{d\alpha (q)}{dq}. \end{aligned}$$

The specific heat reflects the rate of energy variation and can be used to observe the phase transition phenomena. The phase of a thermodynamic system has uniform physical properties, and, here, the phase transition means that certain properties change discontinuously under a critical external condition. The phase transition in the multifractal spectrum has been studied in some simple systems, such as the Cantor set^48^ and logistic map^49^. Here, we find the existence of phase transitions in complex networks, in the multifractal spectrum of the network, the “energy” $\alpha$ has a large fluctuation near $q_{c}$, which can be reflected by the peak of the specific heat $C(q_{c})$. We also give an explanation of the origin of the phase transition from the perspective of the network multifractal structure.

### Network generation model

#### Erdős–Rényi model

Erdős–Rényi (ER) model^30^ proposed by Erdős and Rényi in 1959 is a basic model for generating random networks. It starts from *n* nodes without edges and then chooses each of the possible edges with a certain probability to construct the random network. The model takes in two parameters *n* and *p*, where *n* is the number of nodes in the graph and *p* is the probability of whether to create an edge between two nodes or not. A high *p* value will result in a high average node degree.

The mechanism of ER model includes:

1. Create a network with *n* nodes and no edge.
2. For each node pair (*u*, *v*), with probability *p*, we create an edge between them.

#### Watts–Strogatz model

Watts–Strogatz (WS) model^25^ provides an alternative way to produce a random network. It describes a continuous process of constructing a random network from a regular network by rewiring edges. In the process, we could observe the network with the small-world property, which has a short average length and a high clustering coefficient. To form a WS model, usually, we need three parameters *n*, *k*, and *p*, where *n* stands for the number of nodes in the network, *k* stands for the number of neighbors each node is connected to initially, and *p* stands for the probability of rewiring a chosen edge.

The mechanism of WS model includes:

1. Uniformly place *n* nodes on a ring, and connect each node with its *k* nearest neighbors.
2. For each edge (*u*, *v*) in the network from the previous step, with probability *p*, we rewire this edge to (*u*, *w*), where node w is chosen uniformly from the remaining nodes. (Node *w* will not be chosen if there is already an edge (*u*, *w*)).

#### Barabási–Albert model

Based on the preferential attachment mechanism, Barabási–Albert (BA) model^31^ is considered to generate random scale-free networks. Provided an initially connected graph, we will join more nodes to the graph. Each node will be connected to the existing nodes with probability *p*, where *p* is determined by preferential attachment mechanism regarding each of the existing nodes. The networks following this procedure will be scale-free networks, which means the properties of the networks are immune to the change of their node number and the degree distribution of such networks will follow a power law.

The mechanism of BA model includes:

1. At the initial state, we have an connected graph including $m_0$ nodes.
2. In each of the following state, we add a new node and connect it with *m* $(m\le m_0)$ existing node with probability *p*. The probability $p_i$ (new node connect to node *i*) is
  $$\begin{aligned} p_i=\frac{k_i}{\sum _j {k_i}}, \end{aligned}$$
  where $k_i$ is the degree of node *i* and we sum of all existing node *j*.

## Supplementary Information

Supplementary Information.

## Data Availability

The Ask Ubuntu networks can be accessed from the Stanford Network Analysis Project (SNAP) (https://snap.stanford.edu/data/sx-askubuntu.html). The Facebook-like Forum networks can be accessed from (https://toreopsahl.com/datasets/#online_forum_network). The drosophila brain networks can be accessed from the hemibrain dataset of the FlyEM Project (https://www.janelia.org/project-team/flyem/hemibrain). Source data of the experiments are available at (https://github.com/hlc1209/NMFA_public).

## Code availability

The code and user reference of our methods are available at (https://github.com/hlc1209/NMFA_public).
