# Supplementary material for: Deciphering the generating rules and functionalities of complex networks
Source: Sci Rep. 2021 Nov 25;11:22964. doi: 10.1038/s41598-021-02203-4 (PMC8616909; doi:10.1038/s41598-021-02203-4)
Supplement: Supplementary file 1 — Supplementary Information. [file 41598_2021_2203_MOESM1_ESM.pdf]

Supplementary Materials for Deciphering the Generating Rules and Functionalities of Complex Networks

Xiongye Xiao[1,+], Hanlong Chen[1,+], and Paul Bogdan[1,*]

[1]Ming Hsieh Department of Electrical and Computer Engineering, University of Southern California, Los Angeles, CA 90007, USA.
[*]Correspondence and requests for materials should be addressed to P.B. (email: pbogdan@usc.edu)
[+]these authors contributed equally to this work

ABSTRACT

Network theory helps us understand, analyze, model, and design various complex systems. Complex networks encode the complex topology and structural interactions of various systems in nature. To mine the multiscale coupling, heterogeneity, and complexity of natural and technological systems, we need expressive and rigorous mathematical tools that can help us understand the growth, topology, dynamics, multiscale structures, and functionalities of complex networks and their interrelationships. Towards this end, we construct the node-based fractal dimension (NFD) and the node-based multifractal analysis (NMFA) framework to reveal the generating rules and quantify the scale-dependent topology and multifractal features of a dynamic complex network. We propose novel indicators for measuring the degree of complexity, heterogeneity, and asymmetry of network structures, as well as the structural distance between networks. This formalism provides new insights on learning the energy and phase transitions in the networked systems and can help us understand the multiple generating mechanisms governing the network evolution.

Supplementary Note 1: Description of the regions in the adult drosophila brain.

Medulla (ME): The ME is located near the lobula complex[1]. It is able to obtain direct innervation from photoreceptors. In a more detailed version, the "distal medulla" is responsible for receiving the input signal [2], while the "proximal medulla" will utilize the information from previous layers and do some computation towards those visual signals[3]. The columnar neurons belonging to the medulla interneurons will project outside the ME into the lobula (LO) and lobula plate (LOP) [1].
Lobula (LO): The LO mainly consists of projection neurons. These projection neurons locate between the central brain and the lobula neuropil[3]. The neurons' projections merge at the neck of the LO to form a fiber tract connecting the LO to the central brain[4]. The LO can be further divided into two types of neurons where one is Columnar neurons (LC), receiving signals from 8-9 ommatidia[5, 4] while the other type is tangential and treelike neurons (LT), which receives input from broad visual fields[3].
Lobula plate (LOP): The LOP is located near the lobula plate neuropil, underneath the ME. It is an output neural part in the optic lobe in charging of motion. The lobula plate neuropil can be divided into four layers according to their highest sensitivity towards one direction from front-to-back, back-to-front, up, and down [1, 6].
Ventrolateral neuropils (VLNP, also called optic glomeruli): The VLNP is located right under the optic lobes and is considered to be the visual center in the central brain. It contains 5 regions named by location (anterior optic tubercle (AOTU); anterior ventrolateral protocerebrum (AVLP); posterior ventrolateral protocerebrum (PVLP); posterior lateral protocerebrum (PLP); wedge (WED)). The 5 regions are believed to originate mostly from central brain neuroblasts and are considered to receive and process the visual information from the optic lobes [7, 8].

Supplementary Note 2: Analysis of lethality in protein networks.

Analyzing protein–protein interaction (PPI) network can help us get a better understanding of cell dynamics. Prior research from H. Jeong, et al.[9], have examined an S. cerevisiae PPI network by analyzing latent connections between protein lethality and node degree. The PPI network we investigate here is from P. Uetz, et al.[10], and I. Xenarios, et al.[11], which has 1870 nodes representing different proteins and 2277 interaction links.

H. Jeong, et al. [9] reported that 93% of the total number of proteins have five or fewer links, but only about 21% of them are lethal. By contrast, 0.7% of the yeast proteins have more than 15 links, but 62% or so of these proves lethal. If we examined

the node with degree greater than 15 and less than 30, using node degree centrality, closeness centrality, betweenness centrality, and the node-based fractal dimension (NFD), the results are shown below in Fig. S1, we see that NFD can clearly distinguish between lethal and non-lethal proteins, but the other three centralities cannot. We conclude that Lethal proteins have lower NFD value, and Non-lethal proteins have higher NFD value. The NFD has the potential to determine the relationship between the topological feature and other intrinsic properties of the node.

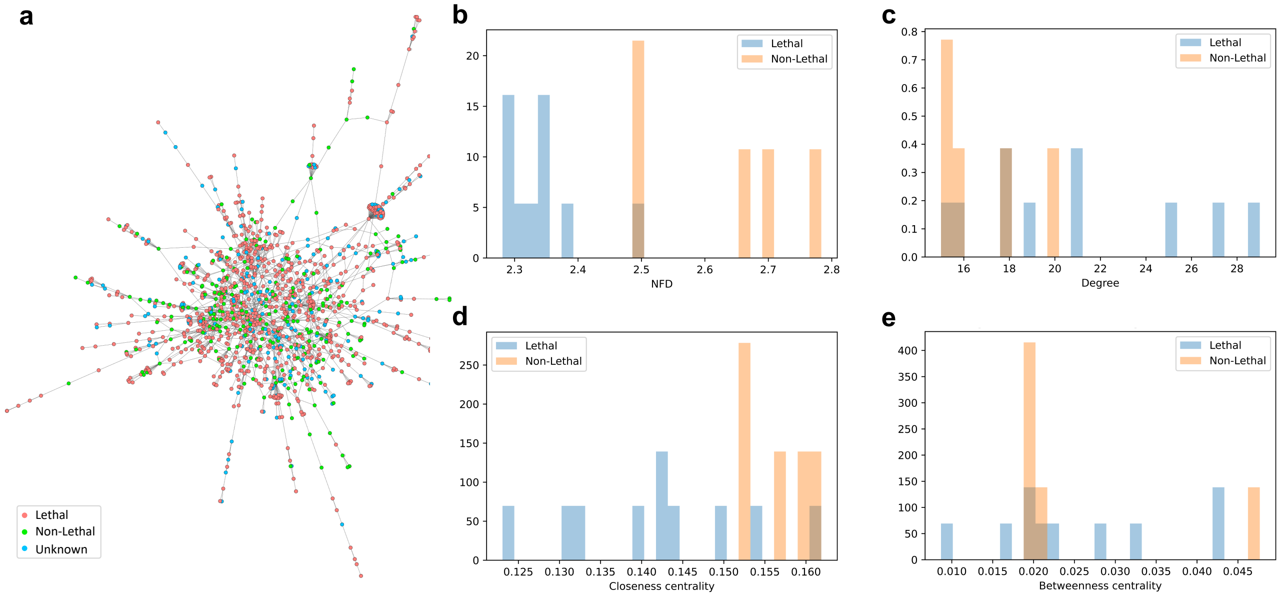

Figure S1. Analysis of lethality on the S. cerevisiae PPI network. a, The protein network where lethal proteins are marked in red, non-lethal proteins are marked in green, and the proteins with unknown lethality are marked in blue. **b, c, d, e,** We present the histograms of the distributions of the number of lethal and non-lethal proteins with the values of NFD **(b)**, degree **(c)**, closeness centrality **(d)**, and betweenness centrality **(e)**.

Supplementary Note 3: NMFA analysis of more real networks.

Here we apply the NMFA on more real unweighted and weighted networks such as typical social networks and neural networks (see Fig. S2). The World-Wide Web (WWW) network[12] is obtained by analyzing the interconnection among web pages under the University of Notre Dame's domain with postfix *nd.edu*. 325729 nodes in the network represent the web pages and 1117563 edges represent links between the web pages. The Email network[13] is obtained by analyzing the email network at the University of Kiel in a period of 112 days. 56576 nodes and 92505 edges represent email address and email communication respectively. From the multifractal spectra and the generalized fractal dimensions of these two social networks (see Figs. S2**a, b, d, e**), we can learn that although the number of nodes and edges of the email network is much smaller than the WWW network, it has higher degree complexity and heterogeneity and higher topological dimension, which means that the connections between users of the email network are more complex and diverse than the WWW network, representing a higher information transmission. Moreover, the multifractal spectrum of the network shows obvious multiscale asymmetry representing the "clump" structure like the Barabasi-Albert network, which implies a preferential attachment generating mechanism. In contrast, the WWW network shows a "thorn" structure. From their specific heat distribution (see Figs. S2**c, f**), we can see that these two networks have different phase transition points, which means different generating mechanisms.

Figs. S2**g-i** show the comparisons of the neural structure network between male and hermaphrodite Caenorhabditis elegans (C. elegans) [14]. The male neural network contains 484 nodes and 1629 edges, while the hermaphrodite one contains 459 nodes and 1445 edges, and both of them are weighted networks. Fig. S2**i** shows that these network shows almost the same specific distribution with the same phase transition point, implying that these two networks of different sexes obey the same generating mechanisms. Even so, we can still capture their structural distances from their multifractal spectra and the generalized fractal dimension (see Figs. S2**g, h**). The male neural network exhibits a little higher degree of complexity and heterogeneity, and a higher fractal dimension than the hermaphrodite one. As has been proved, for the C. elegans, two sexes have some shared neurons, but the male has more sexual specific neurons and muscles than hermaphrodite. This is especially notable in the structure controlling copulation which "consists of 85 male-specific and 64 shared neurons" [14]. This difference in the

complexity and heterogeneity of neural structure can be well illustrated in our method.

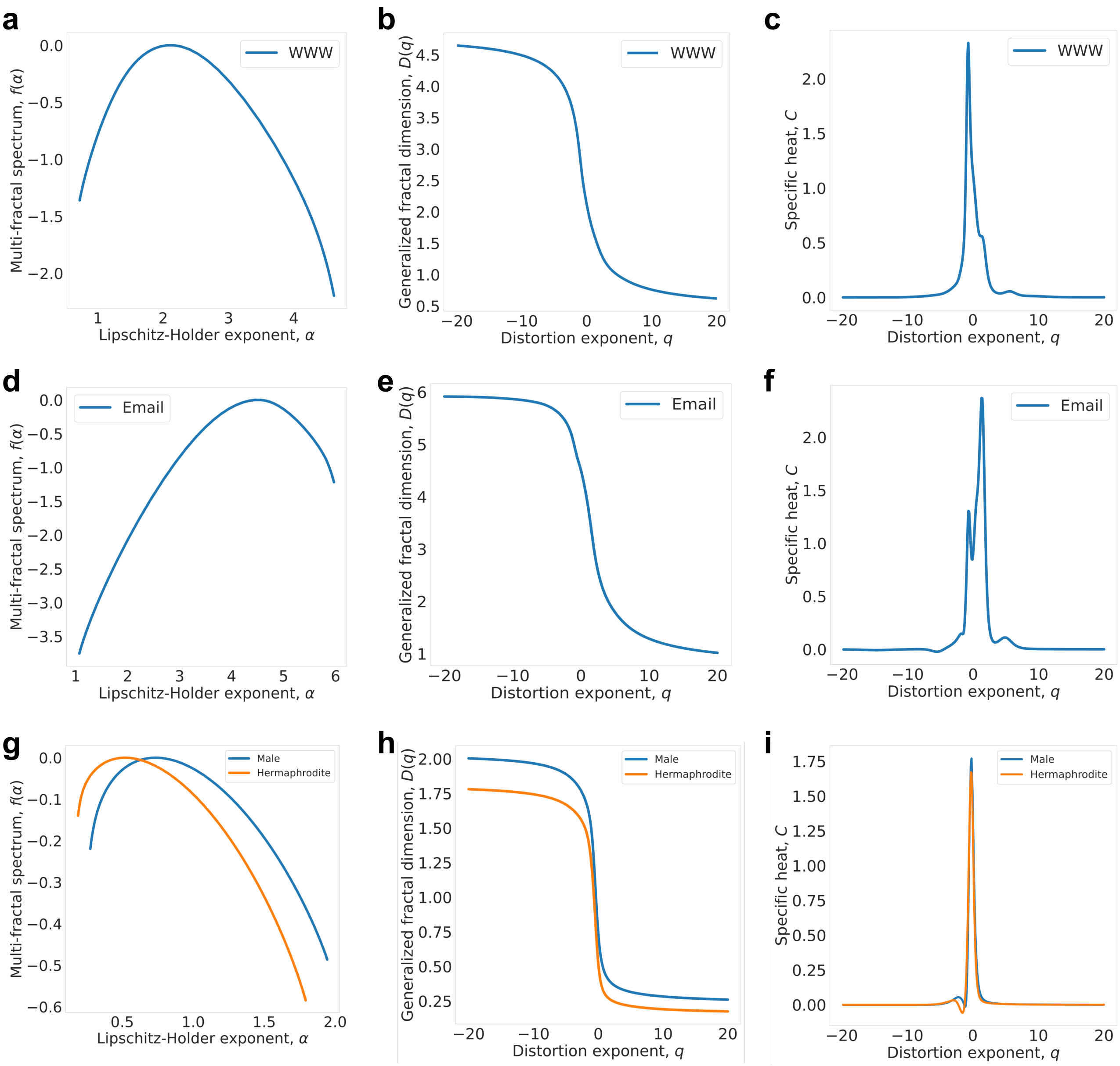

Figure S2. Analysis of real complex networks. a-c, The multifractal spectrum **(a)**, the generalized fractal dimension **(b)** and the specific heat **(c)** of the World-Wide Web (WWW) network. **d-f,** The multifractal spectrum **(d)**, the generalized fractal dimension **(e)** and the specific heat **(f)** of the email network. **g-i,** The comparisons of the multifractal spectra **(g)**, the generalized fractal dimension **(h)** and the specific heat distributions **(i)** of the neural networks of male and hermaphrodite C. elegans.

Supplementary Note 4: Comparison of NMFA and the existing methods.

Different from the traditional multifractal analysis methods[15, 16, 17] based on the renormalization[18] (collectively referred to as MFA in this article), the node-based multifractal analysis (NMFA) framework is the first approach to learn the multifractality of the network based on the node associated feature. The NMFA adopts a different reasoning approach, consisting of the following three innovations: (i) The idea to capture fractal properties for every node in the network: we consider each node in

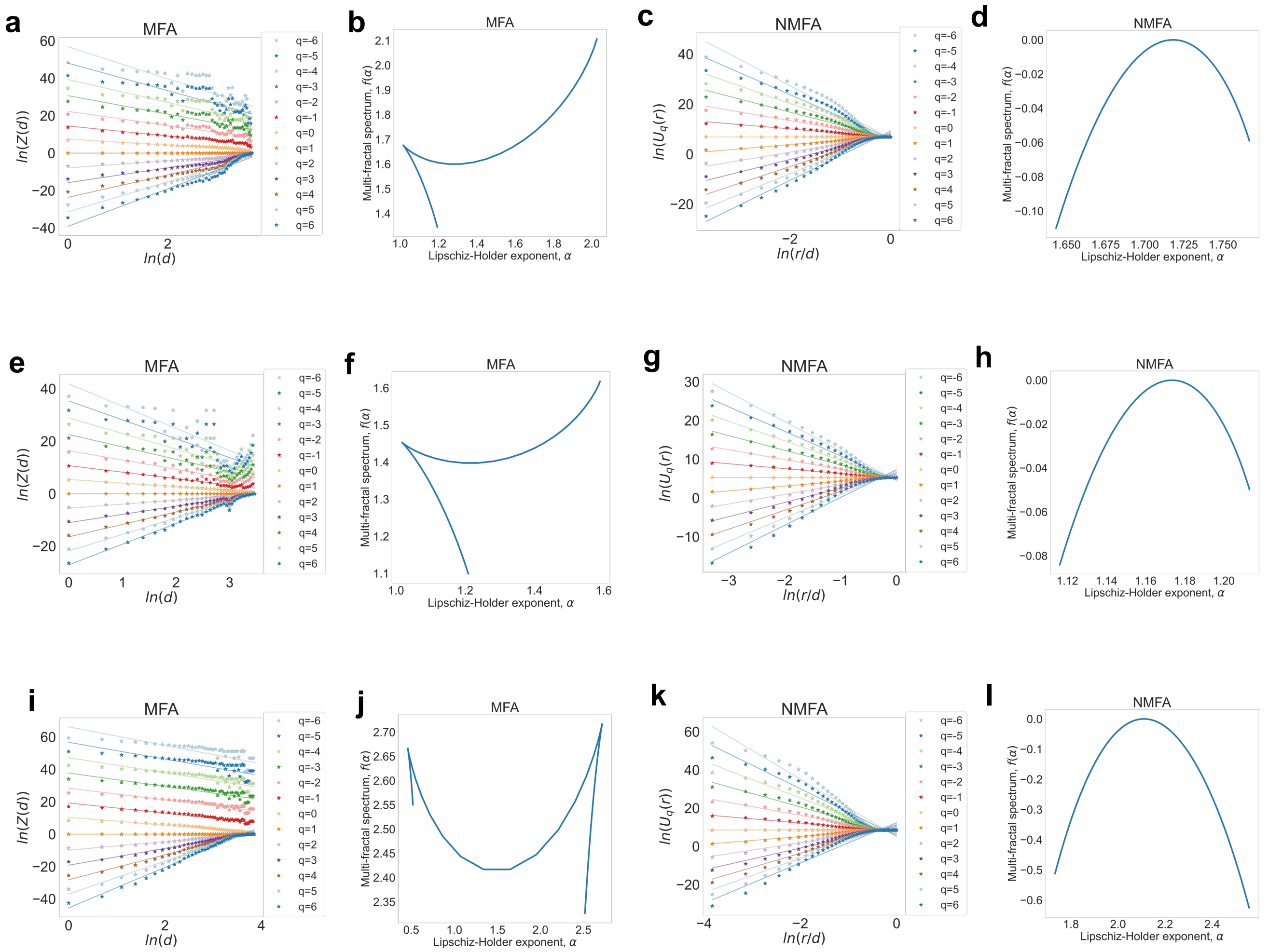

Figure S3. Comparison of NMFA and the existing methods. a-d, We use the Watts-Strogatz model to generate the small-world network with n=1000, k=4, and p=0.02. And we apply the MFA method and our NMFA method on the small-world network to reveal the multifractality. We show the log-log regression of the partition function and box size **(a)**, the MFA spectrum of the network **(b)**, the log-log regression of the partition function and observation scale **(c)**, and the NMFA spectrum of the network **(d)**. **e-h,** We use the Watts-Strogatz model to generate the small-world network with n=1000, k=4, and p=0.02. And we apply the MFA method and our NMFA method on the small-world network to reveal the multifractality. We show the log-log regression of the partition function and box size **(e)**, the MFA spectrum of the network **(f)**, the log-log regression of the partition function and observation scale **(g)**, and the NMFA spectrum of the network **(h)**. **i-l,** We apply the MFA method and our NMFA method on the real network (Power Grid network) to reveal the multifractality. We show the log-log regression of the partition function and box size **(i)**, the MFA spectrum of the network **(j)**, the log-log regression of the partition function and observation scale **(k)**, and the NMFA spectrum of the network **(l).**

the network as the original box and grow it to capture the fractal properties, instead of using boxes to divide the entire network. (ii) The design of the partition function: based on the box-growing method, we integrate the fractal features of all nodes in the network. The distortion factor q is introduced to distinguish the details of different structural features of the nodes in the network. We define the probability measure of node i as $u_i(r) = M_i(r)/M$, where $M_i(r)$ is the number of nodes in box i with radius equal to r, and M is the total number of nodes in the network. Then the partition function (the sum of the qth power of the probability measures) is defined as $U_q(r) = \sum_1^M u_i(r)^q$, and the inspiration for proposing NMFA comes from the discovery that

the partition function has a obvious power-law relationship with the box radius without the renormalization process, which can smoothly help us to quantify the multifractality of the network. (iii) Wider application of multifractal spectrum: the previous multifractal analysis is only suitable for some complex networks with large diameters, not for simple networks or small-world networks, which limits its application. In the contrast, our NMFA method does not have this problem and can be used on universal networks to capture their structural multifractality. Based on this, we also propose the thermodynamics-inspired characterization of the networks and design the structure distance and the multi-scale structural asymmetry.

The most essential difference between our NMFA method and the traditional MFA methods is that the traditional MFA methods are used to observe the self-similarity of the complex networks at different scales based on the renormalization group procedure. In contrast, the proposed NMFA framework devotes to decipher the generating rules of the network by applying the box-growing method on each node to capture the multiple growth rules of the network (which can be characterized by the higher-order relationship between the box mass and the box size) and combining them into the multifractal analysis. This new NMFA framework is applicable to all kinds of networks (e.g., weighted and unweighted, networks of any size and any degree of heterogeneity).

To further illustrate the difference between prior work and our approach, as well as to show the advantages of our NMFA method, we present several examples (i.e., the small-world network to compare these two methods. In the MFA method, the power-law relationship between the partition function ($Z(d)$) and the box size (d) signifies the existence of a fractal property, and the mass exponent τ is defined as $\tau \sim \log Z(d)/\log d$ (see Fig. S3**a**, the value of $\tau(q)$ is the slope of the fitted regression line), then τ is used to calculate the generalized dimension and the multifractal spectrum through Legendre transformation: $\alpha(q) = d\tau(q)/q$ and $f(\alpha) = q\alpha(q) - \tau(q)$.

However, as shown in Fig. S3**a,b,e,f,i,j**, the MFA method cannot provide correct estimates as lots of bias occur when q is negative. The greater the absolute value of q, the greater the bias introduced. In contrast, in our method, this problem can be avoided since the change of the pattern with small probability measures can be accounted for by considering each node in the network as a growing box. Therefore, the relationship between the small probability measure and the observation scale can be captured by the growing box and amplified by the negative q. As shown in Fig. S3**c,d,g,h,k,l**, in our method, the multifractality of the network can be quantified through the log-log relationship between the partition function $U_q(r)$ and the observation scale $\frac{r}{d}$, and the NMFA spectrum can be calculated through the Legendre transformation. We can also use the NMFA spectrum to show the heterogeneous fractal properties of the networks.

Supplementary Note 5: Compare structure distance with other network distance methods.

We compare our structure distance with existing distance methods, i.e., the spectral distance, the correlation distance, and the edit distance.

The spectral distance[19, 20] $d_{spectral}$ between two networks G and G' with the same number of nodes N can be calculated by:

$$d_{spectral} = \sqrt{\sum_{\alpha=1}^{N} [(\mu_\alpha^G - \mu_\alpha^{G'})^2 + (\nu_\alpha^G - \nu_\alpha^{G'})^2]}$$

where μ_α^G and ν_α^G are the αth eigenvalues of the adjacency matrix and Laplacian matrix of graph G, respectively.

Another method to quantify topological difference is the correlation distance[21], which is defined as:

$$d_{correlation} = \frac{1}{2} \sum_{k,k',l} |P_G(k,k',l) - P_{G'}(k,k',l)|$$

where $P_G(k,k',l)$ is the joint probability that randomly chosen two nodes have the degrees k and k' and are separated by the shortest path distance l to each other[21].

The edit distance is a widely accepted method to measure the similarity of two graphs. It is defined as minimum cost of operation sequence transforming G to the isomorphic graph of G'. However, in our experiment shown below, the edit distance cannot be used for networks with more than 15 nodes. Compared to the structure distance we proposed, it has significant longer computing time and more memory consumption.

The structure distance we proposed in this paper is the standard deviation between two generalized dimension D_G, $D_{G'}$ under q, as the formula showed below.

$$d_{structure} = \sqrt{\frac{\int_{q_{min}}^{q_{max}} ((D_G(q) - D_{G'}(q))^2 dq}{q_{max} - q_{min}}}$$

In the table S1, we summarize the time it takes to calculate the different distances on a laptop running at 2.0 GHz frequency, using 32GB DRAM [The source code can be found at https://github.com/hlc1209/Comparison-of-calculation-times-for-different-graph-distances]. Fig. S4**e-g** clearly shows the advantage of the structure distance in terms of time required to complete the computational task. The edit distance is the most time-consuming method, which can hardly be used for real networks with sizes greater than 15. On the contrary, the structure distance we proposed exhibits promising and competitive results for both small-scale and large-scale networks.

Computing time (averaged over 100 times)	The edit distance	The spectral distance	The correlation distance	**The structure distance**
ER model with N=8, p=0.8	1.21s	0.20s	0.01s	0.08s
ER model with N=10, p=0.8	134.20s	0.22s	0.01s	0.09s
ER model with N=15, p=0.8	N/A (out of memory)	0.22s	0.04s	0.10s
ER model with N=100, p=0.8	N/A (out of memory)	0.40s	0.31s	0.47s
ER model with N=500, p=0.8	N/A (out of memory)	6.12s	27.26s	1.50s
ER model with N=1000, p=0.8	N/A (out of memory)	24.41s	198.21s	8.21s

Table S1. Time consumption of calculating different distances

In the main text, we conduct a edge deletion experiment using the Watts–Strogatz network. Here, we provide a simpler example as shown in Fig. S4**a**. In this network, edges (2,6) and (3,5) are the bridge between two communities with the highest edge betweenness centrality and the highest negative (Neg) Ollivier-Ricci curvature. On the contrary, the edges (2,3) and (5,6) have the lowest values of importance. We use the above-mentioned methods to calculate the distance between the network after removing one specific edge and the original network. The results Fig. S4**b** show that only the proposed structure distance (from this manuscript) can capture the difference in deleting the edges with different degrees of importance. Therefore, we believe our proposed structure distance has unique advantages over prior distances.

Supplementary Note 6: Details of the real networks we use in this article.

The numbers of nodes and edges of the real networks we use in this article are shown in Table S2. The Ask Ubuntu networks can be accessed from the Stanford Network Analysis Project (SNAP) [https://snap.stanford.edu/data/sx-askubuntu.html]. The Facebook-like Forum networks can be accessed from [https://toreopsahl.com/datasets/#online_forum_network]. The drosophila brain networks can be accessed from the hemibrain dataset of the FlyEM Project [https://www.janelia.org/project-team/flyem/hemibrain]. The WWW and Email network can be accessed from the datasets of book *Network Science* by *Albert-László Barabási* [http://networksciencebook.com/translations/en/resources/data.html]. The C. elegans neural networks can be accessed from [http://wormwiring.org]. The PPI network can be accessed from KONECT [https://networks.skewed.de/net/interactome_yeast].

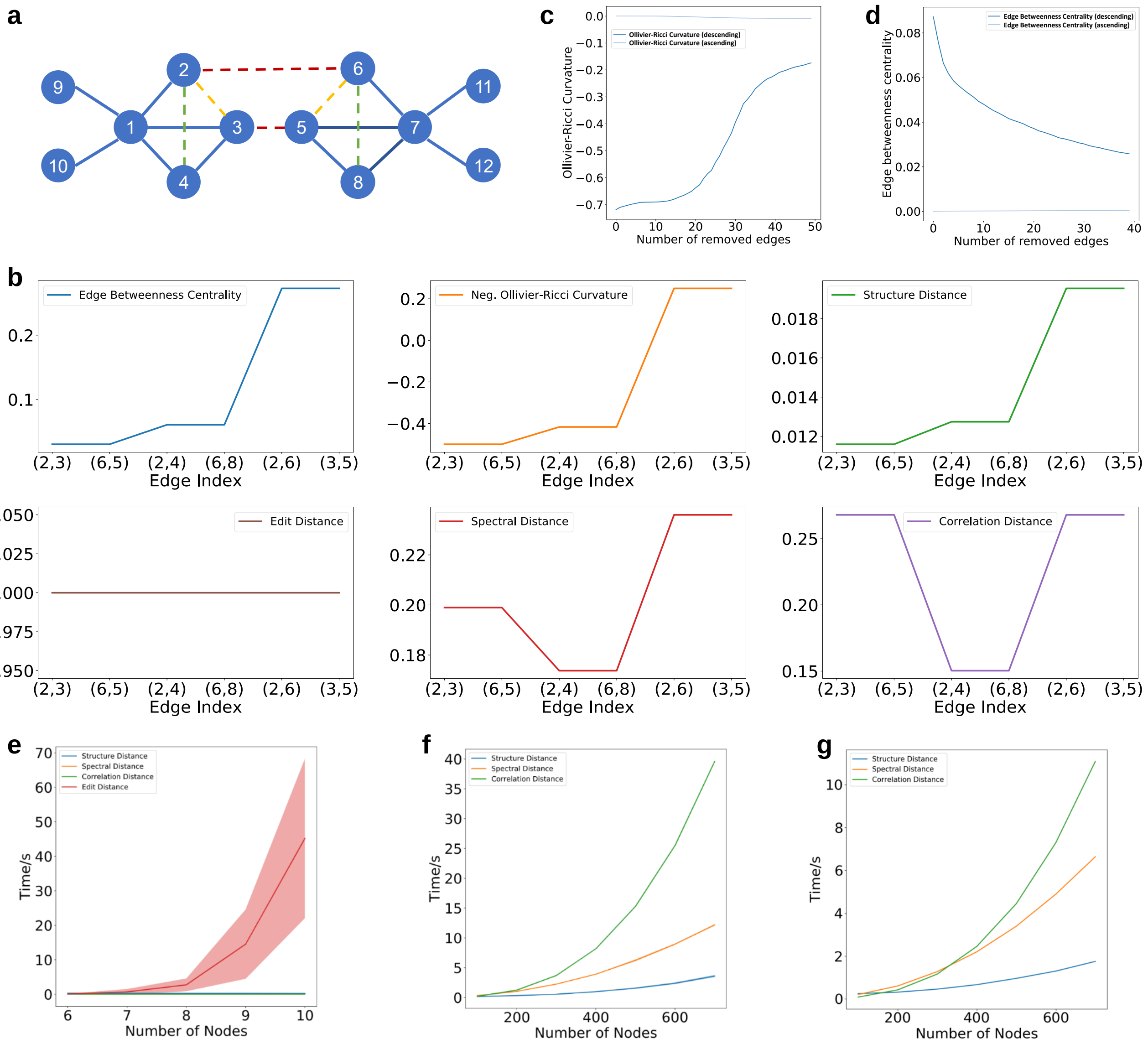

Figure S4. **Compare structure distance with other network distance methods.** In **(a)**-**(d)**, we show the impact of removing critical edges on quantification of the distance between networks according to the spectral, correlation and structure distance strategies. In **(a)** and **(b)**, We remove specific edges in a small network to show the structure distance can demonstrate a similar trend to the value of the Ollivier-Ricci curvature and the edge betweenness centrality. **(c)**-**(d)** The values of edges that were removed in one operation. We use the term "ascending" to denote ascending removal, which means that the least important edges are deleted first. The networks used here are the small-world networks generated by the Watts-Strogatz model with 200 nodes. The experiment was repeated 50 times. The corresponding results of different distance methods are shown in Fig. 1**l-m**. In **(e)**-**(g)**, we show the comparison in terms of time required to compute a distance between two graphs among edit distance, correlation distance, spectral distance and structure distance strategies. The experiment was run 50 times and the curves represent the averaged results. (e) The computational time comparison among the edit distance, the spectral distance, the correlation distance, and the structure distance in a small-scale ER network with p=0.8 linking probability. (f) The computational time comparison among the spectral distance, the correlation distance, and the structure distance in a large-scale ER network with p=0.8 linking probability. (g) The computational time comparison among the spectral distance, the correlation distance, and the structure distance in a large-scale ER network with p=0.3 linking probability.

Table S2. **Details of the real networks**

Network Name	Number of Nodes	Number of Edges	Location
Ask Ubuntu (t0)	77931	232099	Figs. 3**g-i**, **m-o**
Ask Ubuntu (t1)	110942	329853	
Ask Ubuntu (t2)	145656	433098	
Ask Ubuntu (t3)	152597	453211	
Facebook-like Forum (t0)	159	263	Figs. 3**j-o**
Facebook-like Forum (t1)	384	1216	
Facebook-like Forum (t2)	643	3323	
Facebook-like Forum (t3)	899	7046	
LOP	118	202	Fig. 4
ME	122	608	
LO	2264	11573	
AVLP	2975	49093	
PVLP	3210	44999	
AOTU	1370	13965	
PLP	4973	39107	
WED	1954	11956	
OL	2403	12360	
VLNP	9768	148332	
Hemibrain	21300	620785	
PPI	1870	2277	Fig. S1
WWW	325729	1117563	Fig. S2
Email	56576	92505	
Male C. elegans	484	1629	
Hermaphrodite C. elegans	459	1445	

References

[1] K.-F. Fischbach and A.P.M. Dittrich. "The Optic Lobe of Drosophila melanogaster. I. A Golgi analysis of Wild-Type Structure". In: *Cell and Tissue Research* 258.3 (Dec. 1989). ISSN: 0302-766X. DOI: 10.1007/BF00218858. URL: http://www.springerlink.com/content/w360565351565641/ (visited on 05/05/2011).

[2] Javier Morante and Claude Desplan. "The Color-Vision Circuit in the Medulla of Drosophila". In: *Current biology : CB* 18 (May 2008), pp. 553–65. DOI: 10.1016/j.cub.2008.02.075.

[3] Nathalie Neriec and Claude Desplan. "From The Eye To The Brain: Development Of The Drosophila Visual System". In: *Current Topics in Developmental Biology* 116 (Jan. 2016). DOI: 10.1016/bs.ctdb.2015.11.032.

[4] Hideo Otsuna and Kei Ito. "Systematic analysis of the visual projection neurons of Drosophila melanogaster. I. Lobula-Specific Pathways". In: *The Journal of comparative neurology* 497 (Aug. 2006), pp. 928–58. DOI: 10.1002/cne.21015.

[5] John Douglass and Nicholas Strausfeld. "Anatomical organization of retinotopic motion-sensitive pathways in the optic lobes of flies". In: *Microscopy research and technique* 62 (Oct. 2003), pp. 132–50. DOI: 10.1002/jemt.10367.

[6] Matthew Maisak, Juergen Haag, Georg Ammer, et al. "A directional tuning map of Drosophila elementary motion detectors". In: *Nature* 500 (Aug. 2013), pp. 212–6. DOI: 10.1038/nature12320.

[7] Hideo Otsuna and Kei Ito. "Systematic analysis of the visual projection neurons of Drosophila melanogaster. I. Lobula-specific pathways". In: *Journal of Comparative Neurology* 497.6 (2006), pp. 928–958.

[8] Nathalie Nériec and Claude Desplan. "From the eye to the brain: development of the Drosophila visual system". In: *Current topics in developmental biology*. Vol. 116. Elsevier, 2016, pp. 247–271.

[9] Hawoong Jeong, Sean P Mason, A-L Barabási, and Zoltan N Oltvai. "Lethality and centrality in protein networks". In: *Nature* 411.6833 (2001), pp. 41–42.

[10] Peter Uetz, Loic Giot, Gerard Cagney, et al. "A comprehensive analysis of protein–protein interactions in Saccharomyces cerevisiae". In: *Nature* 403.6770 (2000), pp. 623–627.

[11] Ioannis Xenarios, Danny W Rice, Lukasz Salwinski, et al. "DIP: the database of interacting proteins". In: *Nucleic acids research* 28.1 (2000), pp. 289–291.

[12] Reka Albert, Hawoong Jeong, and Albert-Laszlo Barabasi. "Diameter of the World-Wide Web". In: *Nature* 401 (Sept. 1999), pp. 130–131. DOI: 10.1038/43601.

[13] Holger Ebel, Lutz-Ingo Mielsch, and Stefan Bornholdt. "Scale-free topology of e-mail networks". In: *Phys. Rev. E* 66 (3 Sept. 2002), p. 035103. DOI: 10.1103/PhysRevE.66.035103. URL: https://link.aps.org/doi/10.1103/PhysRevE.66.035103.

[14] Steven J. Cook, Travis A. Jarrell, Christopher A. Brittin, et al. "Whole-animal connectomes of both Caenorhabditis elegans sexes". In: *Nature* 571 (2019), pp. 63–71.

[15] Shuhei Furuya and Kousuke Yakubo. "Multifractality of complex networks". In: *Physical Review E* 84.3 (2011), p. 036118.

[16] Stephanie Rendón de la Torre, Jaan Kalda, Robert Kitt, and Jüri Engelbrecht. "Fractal and multifractal analysis of complex networks: Estonian network of payments". In: *The European Physical Journal B* 90.12 (2017), pp. 1–8.

[17] Lázaro Alonso, JA Méndez-Bermúdez, and Ernesto Estrada. "Geometrical and spectral study of β-skeleton graphs". In: *Physical Review E* 100.6 (2019), p. 062309.

[18] Chaoming Song, Shlomo Havlin, and Hernan A Makse. "Self-similarity of complex networks". In: *Nature* 433.7024 (2005), pp. 392–395.

[19] Richard C Wilson and Ping Zhu. "A study of graph spectra for comparing graphs and trees". In: *Pattern Recognition* 41.9 (2008), pp. 2833–2841.

[20] Jiao Gu, Bobo Hua, and Shiping Liu. "Spectral distances on graphs". In: *Discrete Applied Mathematics* 190 (2015), pp. 56–74.

[21] Yuka Fujiki, Shogo Mizutaka, and Kousuke Yakubo. "Fractality and degree correlations in scale-free networks". In: *The European Physical Journal B* 90.7 (2017), pp. 1–9.
